# Effects of *Ocimum sanctum* and *Camellia sinensis* on stress-induced anxiety and depression in male albino *Rattus norvegicus*

**DOI:** 10.4103/0253-7613.70108

**Published:** 2010-10

**Authors:** Imrana Tabassum, Zeba N. Siddiqui, Shamim J. Rizvi

**Affiliations:** 1 Department of Chemistry, J.N. Medical College, Aligarh Muslim University, Aligarh - 202 002, UP, India; 2 Interdisciplinary Brain Research Center, J.N. Medical College, Aligarh Muslim University, Aligarh - 202 002, UP, India

**Keywords:** Anxiety, *Camellia sinensis*, depression, *Ocimum sanctum*, restraint stress

## Abstract

**Objective:**

The aim of this study was to study the ameliorative effects of *Ocimum sanctum* and *Camellia sinensis* on stress-induced anxiety and depression.

**Materials and Methods:**

The study was carried out using male albino rats (200 ± 50 g). The effect of *O. sanctum* and *C. sinensis* was evaluated for anxiety and depression using elevated plus maze (EPM) test, open field test (OFT), forced swim test (FST), and tail suspension test (TST).

**Result:**

Restraint stress (3 h/day for six consecutive days) induced a significant reduction in both the percentage number of entries and time spent in open arms in EPM, and these changes were reversed with post-treatment of aqueous extract of *O. sanctum* and *C. sinensis* (100 mg/kg for 6 days). Restraint stress-induced (a) increased latency and (b) decreased ambulation and rearing were also reversed by *O. sanctum* and *C. sinensis* in OFT. A significant increase in immobility period was observed in FST and TST after restraint stress. *O. sanctum* and *C. sinensis* significantly reduced the immobility times of rats in FST and TST.

**Conclusion:**

*O. sanctum* and *C. sinensis* possess anxiolytic and antidepressant activities.

## Introduction

The influence of environmental conditions such as stress on behavioral and cognitive processes and performance in animal models of psychiatric disorders has been widely investigated.[1] Stress induces a variety of autonomic, visceral, immunological, and neurobehavioral responses such as anxiety, depression, anorexia, and activation of the hypothalamic–pituitary–adrenal axis resulting in elevated corticosterone levels, in animals and humans.[1]

Anxiety is one of the most prominent psychiatric disorders related to a common stress. The elevated plus maze (EPM) is a well-established and widely used animal model of anxiety-like behavior for rodents.[2] The open field test (OFT) is also used frequently. Temporally restricted stressors, such as predator stress in the form of a 5 min exposure to a cat, are potent enough to trigger a state of enhanced and persistent anxiety [Tables 1 and 2].

**Table 1:** Effect of *O. sanctum* and *C. sinensis* in elevated plus-maze test (EPM)

*Groups*	*% Open arm entries*	*% Open arm time*
Control	25.2 ± 3.4	17.3 ± 3.6
RS	12.5 ± 4.3*	6.4 ± 2.4*
RS + OS	27.4 ± 2.0**	18.5 ± 2.8**
OS	26.7 ± 2.4	20.4 ± 2.6
RS + CS	20.3 ± 3.1**	13.6 ± 3.2^a^
CS	22.3 ± 3.6	15.4 ± 1.4

Values are expressed as mean ± S.E.M. (n = 6);

* *P* < 0.05. Statistically significant as compared to control group;

** *P* < 0.01. Statistically significant as compared to RS group.

a *P* < 0.05

**Table 2:** Effect of *O. sanctum* and *C. sinensis* in open field test

*Groups*	*Latency*	*Ambulation*	*Rearing*
Control	2.5 ± 0.7	52.7 ± 5.2	25.2 ± 2.5
RS	4.8 ± 0.3*	32.6 ± 4.6*	10.4 ± 1.3*
RS + OS	2.3 ± 0.5^a^	55.4 ± 3.1**	28.7 ± 3.0**
OS	2.7 ± 0.4	53.6 ± 2.8	26.4 ± 2.2
RS + CS	3.6 ± 0.2**	48.6 ± 4.2^a^	20.3 ± 1.9**
CS	2.8 ± 0.3	53.8 ± 3.6	27.6 ± 3.2

Values are expressed as mean ± SEM (n = 6);

* *P* < 0.05. Statistically significant as compared to control group;

a *P* < 0.05

** *P* < 0.01. Statistically significant as compared to RS group.

Depression is an incapacitating psychiatric ailment that has been estimated to affect 21% of the world population. Chronic stress can induce depressive disorders, and animal stress models are widely used in preclinical antidepressant evaluation.[3] Patients with major depression have symptoms that reflect changes in brain monoamine neurotransmitters, specifically norepinephrine, serotonin, and dopamine.[4] The forced swim test (FST) and the tail suspension test (TST) have been developed to evaluate depression-like behaviors in mice.[5,6] The immobility of the animal in FST reflects a state of “behavioral despair.” The TST also induces a state of despair in animals like that in FST [Tables 3 and 4].

**Table 3:** Effect of *O. sanctum* and *C. sinensis* on immobility period of *R. norvegicus* using tail suspension test (TST)

*Groups*	*Immobility time (s)*
Control	80.73 ± 7.3
RS	150.64 ± 4.1*
RS + OS	78.84 ± 5.7**
OS	82.36 ± 6.4
RS + CS	90.34 ± 6.7**
CS	85.72 ± 4.6

Values are expressed as mean ± SEM (n = 6);

* *P* < 0.001. Statistically significant as compared to control group;

** *P* < 0.05. Statistically significant as compared to RS group.

**Table 4:** Effect of *O. sanctum* and *C. sinensis* on immobility period of *R. norvegicus* using forced swim test

*Groups*	*Immobility time (s)*
Control	60.83±12.4
RS	130.5±15.2*
RS + OS	55.62±10.4**
OS	58.73±9.2
RS + CS	66.73±10.2**
CS	63.21±11.5

Values are expressed as mean ± SEM (n = 6);

* *P* < 0.001. Statistically significant as compared to control group;

** *P* < 0.05. Statistically significant as compared to RS group.

Restraint stress can induce a series of dysfunctions of central nervous system, such as cognitive impairment, anxiety, depression, amnesia, and insomnia. Therefore, close attention was paid to studies of protecting effects of natural potential ingredients on stress-induced injury. Several plants have been investigated, which were once used as tonics due to their adaptogenic and rejuvenating properties in traditional medicine. The drugs of plant origin are gaining increasing popularity and are being investigated for remedies of a number of disorders including antistress adaptogenic activity.[7]

Epidemiological studies have suggested positive association between the consumption of phenolic-rich foods or beverages and the prevention of diseases. These effects have been attributed to antioxidant components such as plant phenolics, flavonoids, and phenylpropanoids among others.[8] *Ocimum. sanctum* and *Camellia sinensis* contain an abundance of naturally occurring polyphenols. Recent studies have shown that polyphenols possess potential effects of neuroprotection.[9] So far, no study has appeared about the protective effects of *O. sanctum* and *C. sinensis* on anxiety and depression induced by restraint stress. Considering substantial neuroprotective properties of *O. sanctum* and *C. sinensis*, we sought to investigate whether *O. sanctum* and *C. sinensis* could improve cognitive impairments induced by restraint stress in rats; and to explore the underlying mechanisms for the same.

## Materials and Methods

### 

#### Ocimum sanctum

Leaves of *O. sanctum* were collected from University campus, identified by a pharmacognist (I.D. No. Husain 1375) and deposited for the record in Aligarh Muslim University (AMU) Herbarium.

#### Camellia sinensis

Leaves of *C. sinensis* were purchased from an authorized dealer, identified by a pharmacognist (I.D. No. Husain 395) and deposited in AMU, Herbarium as a record.

#### Extraction of O. sanctum and C. sinensis

Briefly, the shade dried powder of *O. sanctum* and *C. sinensis* was refluxed for 24 h with double-distilled water (DDW) at 100°C, cooled, and filtered. The solvent was removed under reduced pressure to get the product. The yield of the extracts of *O. sanctum* and *C. sinensis* were 9% and 7% (w/w), respectively, in terms of dried starting material. The residue was stored in the refrigerator until further use. The dose (100 mg/kg) was selected on the basis of preliminary studies that assessed the ability of *O. sanctum* to attenuate the stress-induced alteration in behavioral tests.[10]

#### Animals

Adult male albino rats (200 ± 50 g) were obtained from Central Animal House facility of J.N m0 edical c0 ollege, AMU, Aligarh. The animals kept in polypropylene cages, were housed in air-conditioned room, and maintained on standard pellet diet and water *ad libitum*.

#### Experimental Design

Thirty-six rats were used in this study, and they were divided into six groups. First group treated as control and second was of restraint stress (3 h/day for six consecutive days). Third and fourth groups were treated with the post-treatment of *O. sanctum* and *C. sinensis* (100 mg/kg) aqueous leaf extracts following restraint stress. Fifth and sixth groups were treated with *O. sanctum* and *C. sinensis* alone, respectively. The study was approved by Institutional Animals Ethics Committee (401/CPCSEA).

#### Stress Procedure

**Figure 1:**
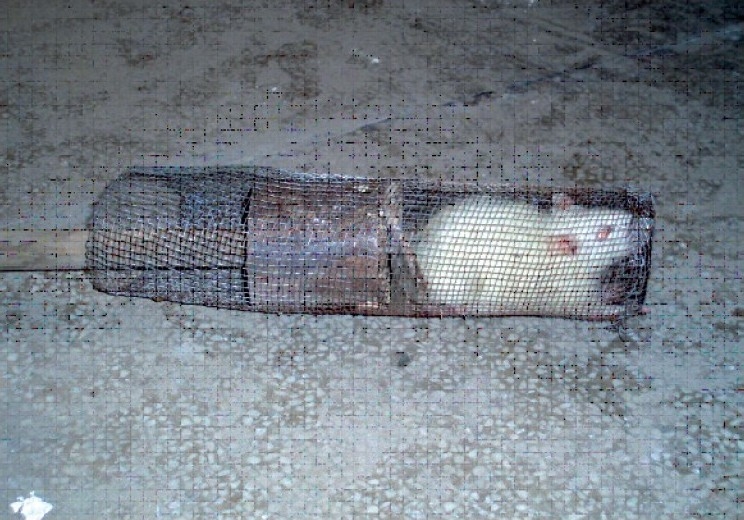
A rat in a wire mesh restrainer.

The animals were subjected to restraint stress in a wire mesh restrainer [Figure 1] for 3 h/day for six consecutive days. The size of restrainers could be adjusted according to the size of the rat ensuring that every rat was immobile. Immediately after the stress procedure, the rats were exposed to behavioral tests.

### Behavioral Tests

#### Elevated Plus Maze Test

This test has been proposed for selective identification of anxiolytic and anxiogenic drugs. The EPM consisted of two open arms and two enclosed arms, 50 × 40 × 40 cm^3^ with an open roof, assayed so that the two arms were opposite to each other. The maze was elevated to a height of 50 cm.[11] After treatment with each group, the rats were placed in the center of the maze, facing one of the enclosed arms. During 5 min test period, the parameters measured were: number of open arm entries and closed arm entries. Subsequently, the percentages of open arm entries and time spent on open arms were calculated from open arm entries and time spent on open arms divided by the total number of entries in both open and closed arms and time spent on open arm exploration divided by total time spent in both open and closed arms, respectively. The procedure was conducted in sound attenuated room.

#### Open Field Test

Locomotor activity was quantified for 5 min in an open field. It was consisted of a square arena 96 × 96 cm^2^ with 60 cm high walls. The walls and the floor were painted in white. The floor was divided in to 16 squares by parallel and intersecting white lines.[12] Four squares defined as the center and the 12 squares along the walls as the periphery. Rats were placed in the very center of the open field and (a) latency, (b) ambulation, and (c) rearing were observed during a 5 min exposure period for both control and treated animals.

#### Forced Swim Test

The test was performed according to a modification suggested by Lucki of the traditional method.[5] The apparatus consisted of a transparent cylinder (50 cm high × 20 cm wide) filled to 30 cm depth with water at room temperature. The water depth was adjusted so that the animals must swim or float without their hind limbs or tail touching the bottom. The duration of immobility was recorded during the last 5 min of the 6 min test swimming session. A rat was judged to be immobile when it floated in an upright position, making only small movements to keep its head above water.

#### Tail Suspension Test

A short piece of paper adhesive tape (about 6 cm) was attached along half the length of the tail (about 3 cm). The free end of the tape was attached to a 30 cm long rigid tape, which hung from a horizontal bar clamped to a heavy laboratory support stand. Suspended animals were surrounded by a white wooden enclosure (40 cm high, 40 cm wide, and 40 cm deep) such that the rat’s head was about 20 cm above the floor. For testing, each rat was suspended by its tail and observed for 6 min. An observer scored the total duration of a passive, “dead weight” hanging (immobility), between the periods of wriggling of the animal to avoid aversive situation.[6]

#### Statistical Analysis

All values are expressed as mean ± SEM. Behavioral data were analyzed using Student’s *t*-test. Significance level was chosen at *P* < 0.001. All statistical analyses were carried out by using SPSS for Windows (SPSS 10.0).

## Results

### 

#### Elevated Plus Maze Test

**Figure 2:**
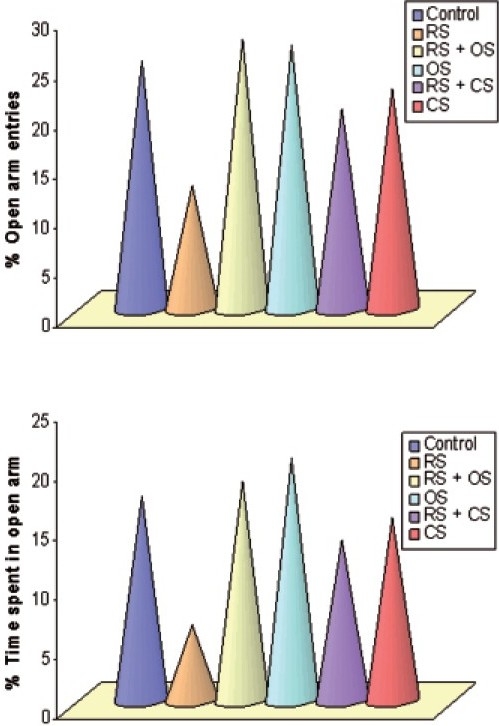
Bar diagram showing the alteration of % open arm entries and % time spent in EPM following restraint stress (3h/day for 6 days) and ameliorative action of *O. sanctum* and *C. sinensis* (100 mg/kg/day for 6 days) on *Rattus norvegicus*.

Analysis of EPM data revealed that restraint stress (3 h/day for six consecutive days) induced a significant reduction in percentage open arm entries by 50% and percentage time spent in open arms by 63% as compared to control (*P* < 0.05). Post-treatment of aqueous extracts of *O. sanctum* and *C. sinensis* (100 mg/kg for 6 days) reversed the restraint stress-induced changes in both open arm entries and percentage time spent in open arms. *O. sanctum* increased the percentage number of open arm entries by 54%, whereas *C. sinensis* by 38%, respectively, as compared to stress group (*P* < 0.01, *P* < 0.05). Percentage time spent in open arms was also increased by 65% and 53% with *O. sanctum* and *C. sinensis* [Figure 2].

#### Open Field Test

**Figure 3:**
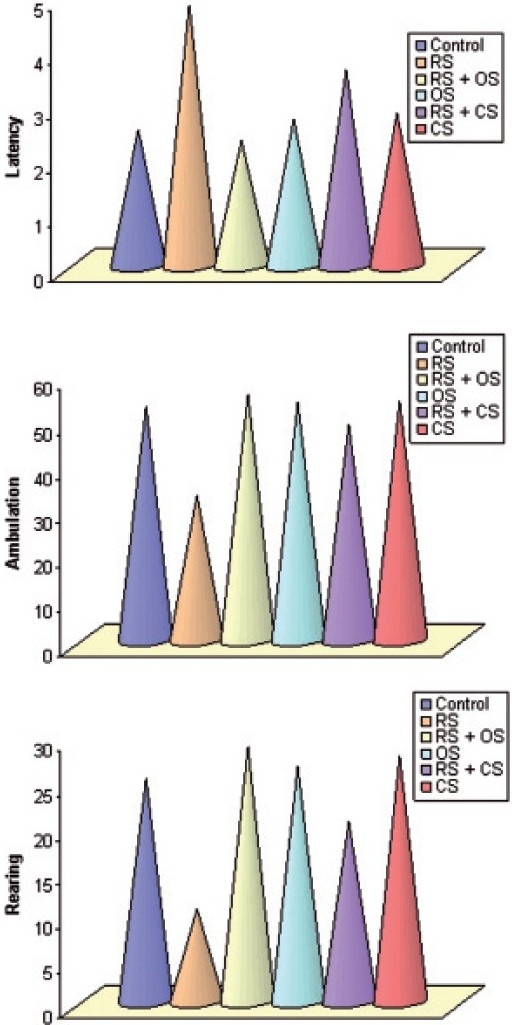
Bar diagram showing the alteration of latency, ambulation, and rearing in OFT following restraint stress (3 h/day for six consecutive days) and ameliorative action of *O. sanctum* and *C. sinensis* (100 mg/kg/day for 6 days) on *R. norvegicus*.

Restraint stress-induced a significant increase in the latency by 48%, decrease in ambulation by 38%, and rearing by 59% as compared to control (*P* < 0.05). Post-administration of *O. sanctum* and *C. sinensis* attenuated the restraint stress effects on the open field behavior. Latency was decreased by 52% with *O. sanctum* and by 25% with *C. sinensis*. Ambulation was increased by 41% and 33% with *O. sanctum* and *C. sinensis*. Rearing activity was also increased by 64% and 49% after the treatment of *O. sanctum* and *C. sinensis*, respectively, as compared to stress (*P* < 0.01, *P* < 0.05) [Figure 3].

#### Forced Swimming Test

**Figure 4:**
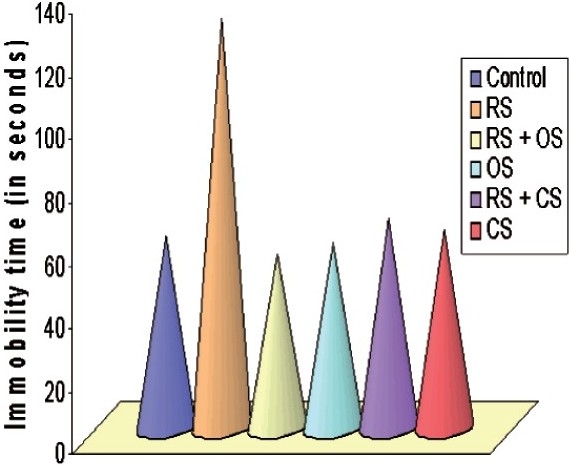
Bar diagram showing the alteration of immobility time in FST following restraint stress (3 h/day for six consecutive days) and ameliorative action of *O. sanctum* and *C. sinensis* (100 mg/kg/day for 6 days) on *R. norvegicus*.

Restraint stress significantly increased the immobility by 53% in the forced swimming test as compared to control (*P* < 0.001). *O. sanctum* caused a 57% reduction in immobility, whereas *C. sinensis* by 49% as compared to control (*P* < 0.05) [Figure 4].

#### Tail Suspension Test

**Figure 5:**
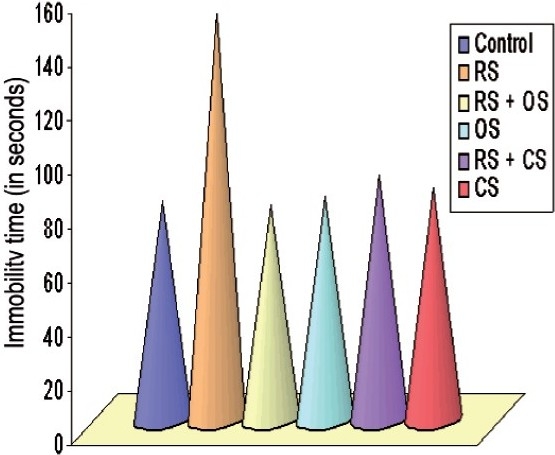
Bar diagram showing the alteration of immobility time in TST following restraint stress (3h/day for 6 days) and ameliorative action of *O. sanctum* and *C. sinensis* (100 mg/kg/day for 6 days) on *Rattus norvegicus*.

Restraint stressed rats exhibited a significant increase in immobility period by 46% as compared to control animals (*P* < 0.001). When *O. sanctum* and *C. sinensis* were administered, significant protection was observed in the anti-immobility effects of *O. sanctum* and *C. sinensis*, i.e. *O. sanctum* decreased the immobility by 48% while *C. sinensis* by 40%, respectively, as compared to stress [Figure 5]. There was no significant change observed in the behavioral tests when *O. sanctum* and *C. sinensis* were given administered.

## Discussion

Environmental factors like stress can influence the neurobehavioral profile of the organism and precipitate an anxiety-like syndrome. Behavioral factors such as emotionality are useful predictors of stress susceptibility.[13] Both EPM and OFT have been used very effectively to assess neurobehavioral profile of animals under the influence of anxiogenic/anxiolytic agents.[3,12] Our results indicated that restraint stress caused a significant reduction in the % number of entries and % time spent in open arms in EPM. Similarly in the OFT restraint stress induced behavioral alterations as evidenced by increase in latency and decrease in ambulation and rearing. These are the indications of high level of fear or anxiety. Our results are in agreement with earlier studies.[14,15]

In the EPM increase in both percentage number of entries and percentage time spent in open arms are indices of anxiolytic activity and our results with aqueous extract of *O. sanctum* and *C. sinensis* were consistent with anti-stress effect of these medicinal plants. Similarly in the OFT, *O. sanctum* and *C. sinensis* decreased latency and increased ambulation and rearing. These behavioral changes were suggestive of decreased fear or anxiety. Thus, *O. sanctum* and *C. sinensis* showed anxiolytic effects against restraint stress. Our results were strongly supported by the study of Gopala Krishna *et al*.[16] who reported anxiolytic activity of NR-ANX-C, a polyherbal preparation, containing aqueous extracts of *Withania somnifera* and *Shilajit* and alcoholic extracts of *O. sanctum* and *C. sinensis*.

*O. sanctum* has been shown to possess cortisol sparing immunostimulant and antioxidant activities. This cortisol sparing immunomodulatory activity of *O. sanctum* may also contribute to the behavioral disinhibitory activity.[17] Earlier studies have shown that *C. sinensis* extract contain many of polyphenolic antioxidants such as catechins, epicatechin, and epigallocatechin gallate. Epicatechin, one of its polyphenolic constituents, has been found to enhance learning and memory ability in mice.[17] Epigallocatechin gallate markedly increased protein kinase C in the membrane and the cytosolic fractions of mice hippocampus the learning site of brain.[18] Oral administration of the aqueous extract of *M. officinalis* in doses ranging from 10 to 100 mg/kg enhanced the exploratory behavior of mice in the EPM and OFT and induced sedation.[19] These studies further support our findings.

The antidepressant activity has been reported from medicinal plants such as *Apocynum venetum* and *Bacopa monniera*.[20,21] The results presented here show, to our knowledge for the first time, that *O. sanctum* and *C. sinensis* given orally is effective in producing significant antidepressant-like effects, when assessed in FST and in TST. Both FST and TST are widely used to screen new antidepressant drugs.[5,6] These tests are quite sensitive and relatively specific to all major classes of antidepressant drugs including tricyclics, 5-HT-specific reuptake inhibitors, MAO inhibitors, and atypicals.[5,6] The FST and TST reflecting a state of despair is reduced by several agents which are therapeutically effective in human depression. This immobility, referred to as behavioral despair in animals, is claimed to reproduce a condition similar to human depression.[6]

The precise mechanisms by which *O. sanctum* and *C. sinensis* produced antidepressant-like effects are not completely understood. Various antidepressant drugs, either by inhibiting MAO-enzyme or by inhibiting reuptake mechanism, increase the central monoamine levels or reverse the stress-induced depressive-like behavior.

Previous studies have shown that noise stress-induced alteration in brain monoamine neurotransmitters (norepinephrine, epinephrine, dopamine, and serotonin). Administration of *O. sanctum* had a normalizing action and controlled the alteration in neurotransmitter levels due to stress.[22,23] Therefore, the antidepressant activity of *O. sanctum* may be correlated with these studies.

*Camellia sinensis* in general as well as its phenolic components catechins and epigallocatechin gallate, have been found to be effective in inhibiting MAO level. Theanine, an amino acid, found in *C. sinensis* has also been found to have beneficial effects by raising the levels of serotonin and dopamine in various important brain regions, particularly the hypothalamus, hippocampus, and striatum.[24] These studies suggest that antidepressant effects might be produced by interaction with adrenoceptors, 5-HT, dopamine receptors, and monoamine oxidase, thereby increasing or decreasing the levels of noradrenaline, 5-HT, and dopamine in the brains of rats. Several herbal preparations that have antidepressant potential also possess antioxidant effects.[25] Thus, antioxidant action may have a role in the mechanism responsible for antidepressant activity. Since *O. sanctum* and *C. sinensis* are antioxidant medicinal plants, the antidepressant activity of *O. sanctum* and *C. sinensis* may be due to their antioxidant activity of these plants.

In conclusion, *O. sanctum* and *C. sinensis* exhibited anxiolytic and antidepressant-like effect in restraint stress-induced anxiety and depression model in rats and these effects may be mediated by the central monoaminergic neurotransmitter system (5-HT and dopamine). In the entire behavioral test, *O. sanctum* shows higher protection than *C. sinensis*. It may be concluded, therefore, that *O. sanctum* is more protective as compared to *C. sinensis* in anxiety and depression.
